# Preservation of hydrogen peroxide-induced oxidative damage in HepG-2 cells by rice protein hydrolysates pretreated with electron beams

**DOI:** 10.1038/s41598-020-64814-7

**Published:** 2020-05-21

**Authors:** Xinxia Zhang, Li Wang, Hui Lu, Zhaoqin Zong, Zhengxing Chen, Yongfu Li, Xiaohu Luo, Yanan Li

**Affiliations:** 1 Key Laboratory of Carbohydrate Chemistry and Biotechnology Ministry of Education State Key Laboratory of Food Science and Technolog, Jiangnan University, Wuxi, 214122 China; 2 National Engineering Laboratory for Cereal Fermentation Technology, Jiangnan University, Wuxi, 214122 China; 3 Jiangsu Provincial Research Center for Bioactive Product Processing Technology, Jiangnan University, LihuRoad1800, Wuxi, 214122 China; 4 Jiangsu Nongken Agricultural Development Co., Ltd., Hengshan Road 136, Nanjing, 210019 China

**Keywords:** Enzyme mechanisms, Peptides

## Abstract

In this paper, electron beam irradiated rice protein hydrolysates (ERPHs) were assessed for their ability to prevent hydrogen peroxide-induced oxidative stress in human HepG-2 cells. The related mechanism was also studied by analyzing the structural changes. Cytotoxicity experiments showed that rice protein hydrolysates pretreated with electron beam irradiation (EBI) were not toxic to cells if appropriate concentrations were applied. Cell viability markedly increased when the cells were treated with ERPHs before H_2_O_2_ induction. Furthermore, the ERPHs effectively suppressed H_2_O_2_-induced ROS production and lipid peroxidation and increased the protein expression levels of the intracellular antioxidant enzymes SOD, GSH-Px and CAT in H_2_O_2_-stressed HepG-2 cells. Consequently, the loss of mitochondrial membrane potential and cell apoptosis was alleviated. Circular dichroism analysis showed that pretreatment of rice protein with EBI significantly changed the secondary structure (the conversion of α-helices to random coils), which is beneficial to the improvement of its antioxidative activity. ERPHs exhibited stronger antioxidative effects than those without irradiation, possibly because of the difference in molecular weight distribution and amino acid composition. These findings indicate an efficient way to produce peptides with better antioxidant activity.

## Introduction

Rice protein (RP) is a byproduct of starch extraction manufacturing. RP has gained much attention recently because of its high yield, high nutritional value and economic value^1^. In addition, RP exhibits hypoallergenic activity^2^ and has a high protein efficiency ratio^3^, which make this product a good protein resource for human nutrition, even as a suitable ingredient of infant food formulations^4^. Although rice protein has many advantages, as mentioned above, the commercial availability of RP in the food industry is restricted by its low solubility and poor functional properties^3^. To overcome these drawbacks, limited enzymatic hydrolysis can be applied. With protease enzymatic modification, the functional properties of food proteins, such as foamability and emulsibility, are improved. Moreover, research has shown that protease-treated rice proteins possess good bioactivities, such as antioxidative properties^5^. In our previous study, the effects of rice protein hydrolysates obtained by various proteases on hydrogen peroxide-induced oxidative stress in HepG-2 cells were investigated. It was found that optimum use of Alcalase could produce peptides with higher antioxidant activity^6^.

In recent years, electron beam irradiation (EBI) has received increasing attention due to its ability to improve food proteins. Previous studies have reported that the application of EBI treatment on proteins has mainly included the following four aspects: (1) functional improvement of proteins^7,8^; (2) promoting crude protein digestibility^9–11^; (3) enhancing the biological activities of proteins^5,12^; (4) facilitating the proteolytic effect via the modification of the protein structure^13^. Specifically, EBI treatment could produce polypeptides with a variety of biological activities, such as antioxidant capacity. Lin *et al*. noted that EBI pretreatment significantly increased the DPPH radical scavenging capacity of hydrolyzed corn protein. It was found that the microcosmic surface structure of corn protein was indeed changed by EBI. Rupture of the particle structure increased the contact area between the protein granules and the enzyme. This rupture should facilitate a more effective release of antioxidative peptides, which enhanced antioxidant activity^12^. In addition, Wang *et al*. demonstrated that the antioxidant activity of pea protein hydrolysates was effectively improved by the pretreatment of pea proteins with EBI. This improvement may be attributable to protein unfolding and scission induced by the energy generated by an electron beam accelerator. Such changes in protein structure could expose more peptide bonds to proteolytic enzymes, which may contribute to an increase in antioxidant activity^5^. The modification of these protein hydrolysates could promote their application in food industries as antioxidants. Thus, it is important to explore the influence of EBI treatment on the antioxidant activity of rice protein hydrolysates (RPHs). However, to our knowledge, there is no report on the effect of electron beam irradiation treatment on the structure and antioxidant activity of rice protein hydrolysates.

Therefore, the objectives of this research were to explore the impact of EBI treatment on the antioxidant activity of RPHs by cell-based bioassays, including cytotoxicity and cell viability, intracellular ROS level, antioxidant enzyme activity, apoptosis, and related mechanisms. Rice protein hydrolysates without EBI treatment are expressed by NRPHs (non-irradiated rice protein hydrolysates). The samples pretreated with EBI are expressed by ERPHs (EBI-treated rice protein hydrolysates). Both enzymes are RPHs. The results of this study are expected to provide a theoretical basis for the development of the electron beam irradiation technique and provide technical support for further research on new antioxidant peptides.

## Methods and materials

### Materials

Rice protein (82.9% protein, 7.9% water, 1.35% ash, 6.25% lipid, 1.60% sugar) was provided by Shanyuan Biotechnology Co. Ltd. (Wuxi, China). Alcalase (2.4 L) was purchased from Novozymes (Beijing, China). HepG-2 cells were purchased from the Institute of Biochemistry and Cell Biology, SIBS, CAS (Shanghai, China). Dulbecco’s Modified Eagle’s medium (DMEM), fetal bovine serum (FBS), and other cell culture materials were purchased from Gibco BRL, Life Technologies (USA). Cell Counting Kit-8 (CCK-8), a reactive oxygen species assay kit, malondialdehyde (MDA), superoxide dismutase (SOD) and glutathione peroxidase (GSH-Px) assay kits, an annexin V-FITC apoptosis detection kit, and a mitochondrial membrane potential assay kit with JC-1 were all obtained from Beyotime Biotechnology Co. Ltd. (Shanghai, China). A reactive oxygen species assay kit was purchased from the Nanjing Jiancheng Bioengineering Institute (Nanjing, China). These and all other chemicals and reagents were of analytical grade or higher.

### Electron beam treatment

Electron beam irradiation was conducted by the AIBANG EB-Tech Co., Ltd., Wuxi, China, using a high-energy linear accelerator. Rice protein was irradiated in the powder form. One hundred grams of the protein was sealed in a sterile food-grade polyethylene plastic bag, and the thickness was approximately 1.0 cm. Then, rice protein was irradiated with varying doses (5 kGy, 10 kGy, 20 kGy, or 30 kGy, the irradiation depended on the exposure time) by electron beam irradiation at 5.0 MeV. All irradiations were performed at room temperature. After irradiation, the samples were stored at -20 °C until further use.

### Enzymatic hydrolysis of RP

The RP was stirred in distilled water (5% [w/v]) for 30 min at 55 °C, pH 8.5. The reactions were then carried out with Alcalase 2.4 L for 2 h. The enzyme to substrate (E/S) ratio was 1:100 (w/w), and 1 M NaOH was applied to maintain the pH level of the slurry constant. Two hours later, the enzyme was inactivated by boiling in a water bath for 10 min. After cooling, the hydrolysates were adjusted to a pH level of 7.0 and centrifuged at 10 000 g for 20 min. The supernatant was then freeze-dried and stored at -20 °C for further use.

### Cell culture and treatment

Human HepG-2 cells were propagated in a DMEM nutrient mixture supplemented with 10% FBS, 100 units per mL penicillin, and 100 μ g per mL streptomycin at 37 °C in an incubator with 5% CO_2_ and 95% air. All experiments were carried out at 12 h after the cells were seeded onto microplates. HepG-2 cells were incubated with RPHs for 48 h, and then 0.4 mM H_2_O_2_ was added, and the cells were incubated for another 4 h.

### Determination of cell cytotoxicity and viability

Cytotoxicity assay: HepG-2 cells were seeded onto 96-well plates at a density of 4 × 10^5^ cells per mL and incubated at 37 °C in a CO_2_ incubator overnight. The cells were incubated with 100 µL of complete culture medium containing 0.2, 0.4, 0.6, 0.8, and 1.0 mg mL^1^ of RPHs for 72 h. Cell cytotoxicity was determined by adding 10 µL CCK-8 to each well and incubated for 4 h. The absorbance was measured at 450 nm by a microplate reader (M5, Molecular Devices, USA). Cell cytotoxicity was expressed as the following equation:

$${\rm{Cell}}\,{\rm{cytotoxicity}}=({{\rm{A}}}_{{\rm{control}}}-{{\rm{A}}}_{{\rm{treated}}})/{{\rm{A}}}_{{\rm{control}}}\times 100 \% $$

Cell viability: HepG-2 cells were seeded onto 96-well plates at a density of 4 × 10^5^ cells per mL and incubated at 37 °C in a CO_2_ incubator overnight. The cells were incubated with 100 µL of complete culture medium containing 0.2, 0.4, 0.6, 0.8, 1.0 mg mL^1^ of RPHs or 0.08 mg mL^1^ of VC for 48 h. After removal of the medium, 100 µL H_2_O_2_ (0.4 mM) was added to each well and incubated for another 4 h. Cell viability was determined by adding 10 µL CCK-8 to each well and incubated for 4 h. The absorbance was measured at 450 nm by a microplate reader (M5, Molecular Devices, USA). Cell viability was expressed as the following equation:

$${\rm{Cell}}\,{\rm{viability}}={{\rm{A}}}_{{\rm{treated}}}/{{\rm{A}}}_{{\rm{control}}}\times 100 \% $$

### Measurement of intracellular reactive oxygen species (ROS)

A reactive oxygen species assay kit was used to determine the level of ROS. Briefly, the cells were treated with RPHs (0.8 mg mL^-1^) for 48 h and exposed to 0.4 mM H_2_O_2_ for 4 h. After treatment, the cells were further incubated with 10 μM DCFH-DA at 37 °C in the dark for 30 min. Subsequently, the cells were analyzed for DCF fluorescence by a laser scanning confocal microscope (LSM 710, Carl Zeiss AG, Germany). The relative DCF fluorescence was provided directly by the apparatus.

### Measurements of SOD, GSH-Px, CAT and MDA

The activities of superoxide dismutase (SOD), glutathione peroxidase (GSH-Px), catalase (CAT) and the content of malondialdehyde (MDA), a product of lipid peroxidation, were measured by assay kits (Beyotime Biotechnology Co., Ltd, Shanghai, China). All procedures completely complied with the manufacturer’s instructions. One unit of SOD activity was defined as the inhibition rate when the above response reached 50%. One unit of GSH-Px was defined as 1 μmol NADPH oxidized in 1 min. The CAT assay was based on its ability to scavenge H_2_O_2_. The content of MDA was determined by measuring the absorbance of MDA-TBA from the reaction between MDA and TBA at 450 nm.

### Cell apoptosis and mitochondrial membrane potential (MMP)

HepG-2 cells were cultured in 6-well plates and allowed to attach overnight. The cells were treated with RPHs (0.8 mg mL^-1^) for 48 h and exposed to 0.4 mM H_2_O_2_ for 4 h. Then, the cells were collected, washed and resuspended in PBS.

Apoptosis assay: To evaluate the apoptosis of the cells, an annexin V-FITC apoptosis detection kit was used. The cells treated above were stained with 5 μL annexin V-FITC and 10 μL propidium iodide (PI) for 15 min at room temperature in the dark. The cells were analyzed on a flow cytometer (FACSCalibur, Becton Dickinson, USA).

MMP assay: A mitochondrial membrane potential assay kit with JC-1 was used to examine the MMP. The cells collected above were incubated with 0.5 mL of a JC-1 working solution for 20 min at 37 °C in the dark, washed twice with a JC-1 staining buffer and resuspended in 0.5 mL of PBS. Flow cytometry (FACSCalibur, Becton Dickinson, USA) was used to analyze the cells.

### Fourier transform infrared spectroscopy (FTIR) analysis

FTIR of the sample was determined according to a previously published method^14^ with slight modification. FTIR spectroscopy was recorded using a Nicolet iS10 FTIR spectrometer (ThermoFisher Scientific, Marietta, OH, USA). Two milligrams of protein powder was mixed with KBr, ground, and then pressed into a pellet. The spectra were measured in a wavenumber range of 4000–400 cm^-1^ at a 2 cm^-1^ resolution. Omnic V8.1 (ThermoFisher Scientific, USA) and PeakFit 4.12 (SeaSolve Software Inc., USA) were used to determine the α-helix, β-sheer, turns and unordered structure percentages in the protein mixtures.

### Circular dichroism (CD) spectra analysis

Far-UV CD spectra of RPs were recorded using a MOS-450 spectrometer (BioLogic Science Instruments, Ltd., Claix, France). RPs were dissolved in 0.01 M PBS (pH 8.0) at a concentration of 0.5 mg/mL. A 0.1 cm path length quartz cell and a 0.2 nm band were used for the measurement. The content of the protein secondary structure was calculated by protein two and three structure analysis software CDPro.

### Determination of molecular weight distribution

The molecular weight distribution of the RPHs was examined by gel permeation chromatography using an HPLC system (1260 Infinity, Agilent Technologies, USA). A TSKgel2000 SW XL column (7.8 mm i.d. × 300 mm; Tosoh, Tokyo, Japan) was equilibrated with the following mobile phase: aceto-nitrile/water/trifluoroacetic acid was 45/55/0.1 (v/v) at a flow rate of 0.5 mL min^-^1. A molecular weight calibration curve was prepared using the following protein standards: cytochrome C (12.5 kDa), bacitracin (1450 Da), tetrapeptide GGYR (451 Da), and tripeptide GGG (189 Da) (Sigma St. Louis, MO, USA).

### Amino acid analysis

Two hundred milligrams of RPHs were digested in sealed, evacuated glass tubes with 6 M HCl at 110 °C for 24 h. After being evaporated under nitrogen at 60 °C, the hydrolysates were diluted with water to 100 mL and then filtered. The amino acid analysis of the filtrate was performed using an automatic amino acid analyzer (L-8800, Hitachi, Japan).

### Statistical analysis

Data were analyzed using IMB SPSS statistics 2.0 software. The differences between the mean values of the samples were determined using the least significant difference (LSD) test at a level of 0.05.

## Results and Discussion

### Effects of EBI treatment on the cellular antioxidative capacity of RPHs and the related mechanism

#### Cell cytotoxicity and viability

**Figure 1:**
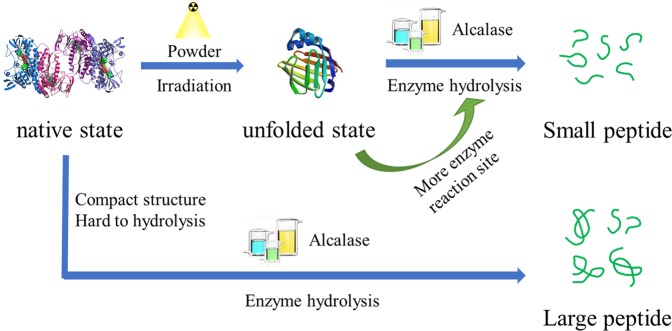
Schematic illustration of the preparation of bioactive peptide with or without EBI irradiation.

**Figure 2:**
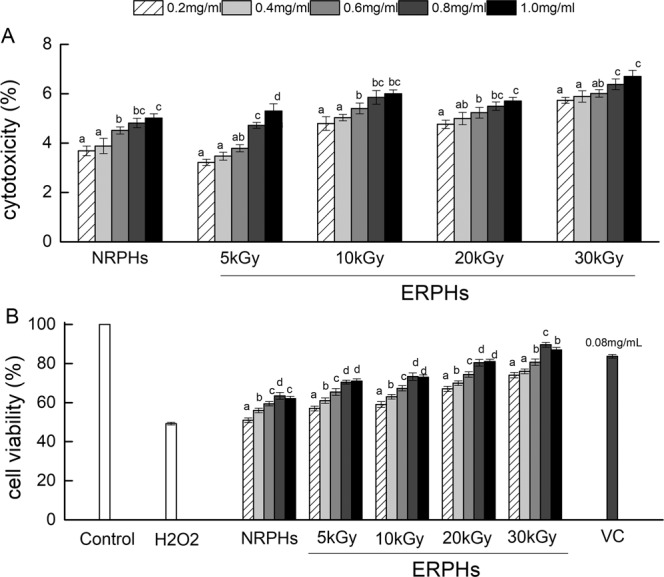
(**A**) The cytotoxic effects of ERPHs on HepG-2 cells. Cells were co-cultured with NRPHs and ERPHs for 72 h and measured by CCK-8 analysis. (**B**) Proliferative effects of ERPHs and VC on HepG-2 cells. Cells were pre-incubated with NRPHs and ERPHs for 48 h prior to treatment with 0.4 mmol L^-1^ H_2_O_2_ for 4 h. After the treatment, cell viability was determined by CCK-8 analysis. Data are shown as means ± S.D.

In this study, a reliable *in vitro* cellular model, established in our previous study^6^, was used to investigate what occurs in the human body more accurately. First, a CCK-8 assay was used to examine whether the samples alone would cause cell death. As shown in Figs. 1 and 2A, the cytotoxicity levels of all samples, with or without irradiation pretreatment, were all within 10%. Furthermore, the cytotoxicity levels increased slightly with increasing irradiation dose. The results indicated that the concentration used in this study was not toxic to the cells^15^. Therefore, the inhibitory activity of NRPHs (nonirradiated RPHs) and ERPHs (EBI-treated RPHs) in H_2_O_2_-induced cellular stress in HepG-2 cells, which would be shown in the following study, was not due to toxicity. This outcome also suggested that EBI pretreatment of RPHs was safe for cells if a suitable concentration was applied.

Then, the proliferative effects of ERPHs on HepG-2 cells were examined. The cells were preincubated with NRPHs and ERPHs for 48 h prior to treatment with 0.4 mmol L^-1^ H_2_O_2_ for 4 h. As shown in Fig. 2B, the cell viability of HepG-2 cells markedly increased when the cells were pretreated with RPHs before H_2_O_2_ induction, compared to the cells treated with H_2_O_2_ alone (P < 0.05). The protective activity was more pronounced when the cells were pretreated with various ERPHs at the same concentration. Moreover, the sample concentration affected the cell viability of HepG-2 cells. The cell viability increased gradually as the concentration increased from 0.2 mg mL^-1^ to 0.8 mg mL^-1^ but decreased when the concentration continued to increase. Therefore, we chose 0.8 mg mL^-1^ for the following experiment. Additionally, the inhibitory effect against H_2_O_2_-induced cytotoxicity increased with increasing irradiation dose. The irradiation of rice protein at 30 kGy resulted in the strongest inhibitory effect of the hydrolysates against the injury induced by H_2_O_2_, with cell viability values 41.29% higher than those for NRPHs (0.8 mg mL^-1^). The data indicated that pretreating rice protein with EBI had the potential to facilitate a protective effect of the hydrolysates against H_2_O_2_-induced injury in cells. The reason for this phenomenon was that the structure of EBI-treated rice proteins may be broken, exposing more activity sites that are susceptible to enzyme attack, and this effect may lead to the generation of a wide variety of smaller peptides and free amino acids^16^, which are key facts that can significantly improve the antioxidant properties of hydrolysates^17^. The peptides irradiated at 30 kGy, which had the strongest antioxidant activity in the ERPHs, may contain more smaller peptides and free amino acids. On the other hand, although the antioxidant activity of peptide is lower than that of VC, the bioactive peptide, which contains a variety of amino acids, is not only an antioxidant but also a nutritional supplement. Moreover, the peptide is naturally derived and safer than chemically synthesized antioxidants. Therefore, peptides have their own unique advantages to be a natural antioxidant compared with conventional chemical antioxidants.

#### Reactive oxygen species (ROS)

**Figure 3:**
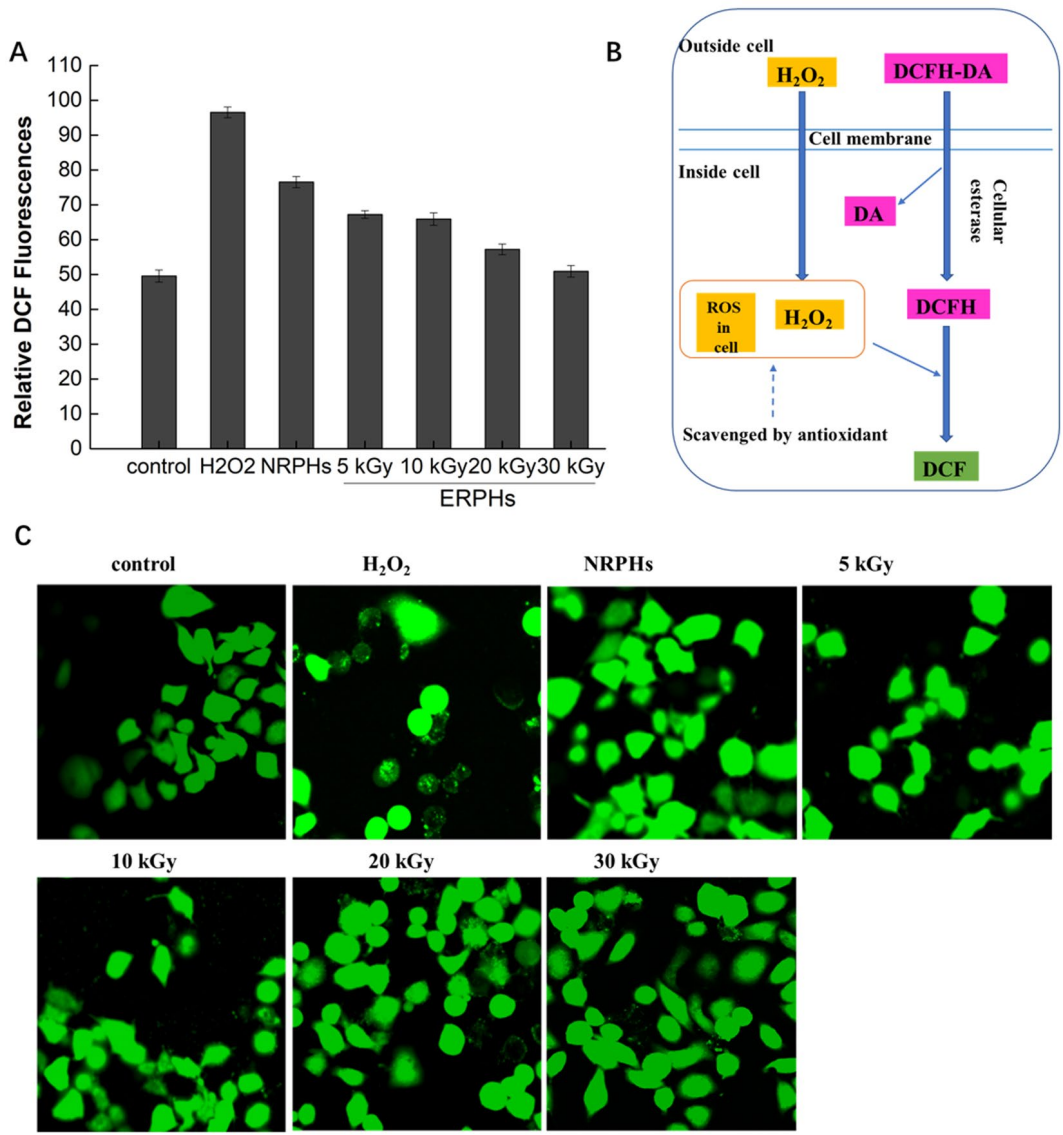
Effect of ERPHs on the intracellular ROS level. HepG-2 cells were pretreated with NRPHs and ERPHs for 48 h before treatment with 0.4 mM H_2_O_2_ for 4 h. Then the cells were exposed to DCFH-DA for 30 min. DCF fluorescence of the treated cells were measured by using a laser scanning confocal microscope. Data are shown as means ± S.D.

To determine whether the protective effect of rice hydrolysates on cells is related to their antioxidant activities, the production rates of ROS in cells were assessed by a DCFH-DA fluorescent probe. The ROS in the cells could oxidize the intracellular DCFH to fluorescent DCF. Antioxidants prevented the oxidation of DCFH and reduced the formation of DCF (Fig. 3B). As shown in Fig. 3A, the DCF fluorescent signal liberated in cells for each of the hydrolysate-protected groups was considerably lower than that in the H_2_O_2_-induced group (p < 0.05). This result supports the hypothesis that the cytoprotective effect of RPHs may be associated with their antioxidant capacities. In particular, treatment with ERPHs resulted in significantly weaker DCF fluorescence intensity compared with that of the NRPH-treated group, suggesting that the administration of ERPHs had a better inhibitory effect on ROS generation in HepG-2 cells under oxidative conditions. Furthermore, the fluorescence level of the ERPH-protected group decreased with increasing irradiation dose. The irradiation of rice protein at 30 kGy resulted in the lowest fluorescence signal, showing the highest scavenging activity of intracellular ROS. The images of the HepG-2 cells treated with NRPHs and ERPHs demonstrated the same result (Fig. 3C). The average brightness of the ERPH-treatment groups tended to be more muted than that of the groups pretreated with NRPHs. These results provide evidence that the EBI pretreatment of rice protein can effectively enhance the antioxidant effect of hydrolysates and increase the inhibition of H_2_O_2_-induced cell damage.

In general, excess H_2_O_2_ can induce the intracellular accumulation of ROS in HepG-2 cells and disrupt the balance between the production of ROS and cellular defense, causing stress injury^18^. Furthermore, the overgeneration of ROS would damage biological molecules, such as proteins and lipids, and cause a loss of cell functioning, which would lead to cell apoptosis^19,20^. However, pretreatment with RPHs dramatically abrogated these negative impacts by reducing the ROS levels in the cells. Interestingly, when the cells were pretreated with the same amount of added hydrolysates, ERPHs were more efficiently antagonized DCFH oxidation than NRPHs, supporting their cellular ROS scavenging effect. The difference between NRPHs and ERPHs might be because EBI treatment of rice protein enhanced the proteolytic effect via the modification of the protein structure, leading to a release of antioxidative amino acids and a change in ROS scavenging ability, as previously reported^5,6^. In addition, protein breakdown also resulted in the production of low molecular weight polypeptides, which also contributed to the enhancement of their antioxidability, facilitating the ability to enter cells and play a protective role^21^.

#### Activity of antioxidant enzymes and lipid peroxide levels

**Figure 4:**
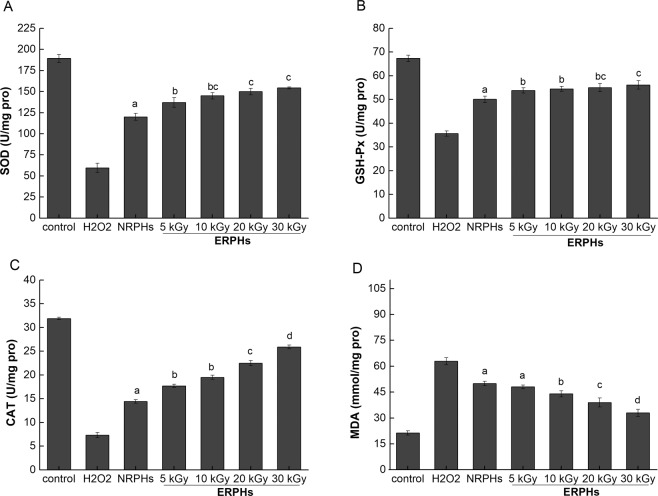
The effect of ERPHs on SOD, GSH-Px, CAT and MDA activity in H_2_O_2_-treated HepG-2 cells. NRPHs and ERPHs were added to the culture 48 h prior to H_2_O_2_ addition, then cells were incubated with 0.4 mM H_2_O_2_ for 4 h. Data are shown as means ± S.D.

Intracellular ROS levels increased sharply when H_2_O_2_ was added, causing oxidative damage in the cells (Fig. 3A). Nevertheless, this damage could be inhibited by the antioxidant system of the cell to some extent. Several enzymes play a pivotal role in eliminating ROS and defending against H_2_O_2_-induced damage, including SOD, GSH-Px and CAT^22^. As shown in Fig. 4A, the treatment of HepG-2 cells with H_2_O_2_ caused a decrease in the intracellular SOD level by 68.50%, while preincubation of the cells with RPHs markedly attenuated this decrease. Moreover, the effect of ERPHs on the expression of SOD was higher than that of NRPHs, in a gradual manner as the irradiation dose increased, reaching a maximum of 61.35% recovery effect at 30 kGy (treatment with NRPHs only restored 50.29%). GSH-Px and CAT activity also significantly increased when the HepG-2 cells were treated with RPHs and more remarkably with ERPHs (P < 0.05) compared with that in the H_2_O_2_-induced group. At 30 kGy of ERPHs, the H_2_O_2_-induced decrease in GSH-Px and CAT activities was restored and the activity increased to 56.13 ± 1.78 and 25.9 ± 0.39 U/mg pro, respectively, which was higher than that recovered by NRPHs (Fig. 4B,C).

MDA, a sensitive indicator of the peroxidation of cellular lipids in cells^23^, was also detected to identify the protective effect of antioxidative RPHs. Figure 4D shows that the MDA content in cells subjected to H_2_O_2_ stress increased nearly 3-fold when compared with the control group. However, the addition of RPHs significantly inhibited the formation of MDA (P < 0.05). Again, the RPHs pre-irradiated by EBI showed a better inhibitory effect than the NRPH-treated group, with the MDA content decreasing by 47.49% at 30 kGy, compared to that in the H_2_O_2_-induced group. The addition of the remaining four hydrolysates (NRPHs, ERPHs pretreated at 5 kGy, 10 kGy, and 20 kGy) reduced the content of MDA by 20.43%, 23.62%, 29.98% and 37.94% when compared to that in the group treated with H_2_O_2_ alone.

Excessive ROS can cause marked irreversible damage to cells and eventually lead to apoptosis^24^. The data shown above indicated that the pretreatment of cells with RPHs can stimulate the intracellular antioxidant system, promote the expression of antioxidant enzymes, and protect cells from H_2_O_2_-induced injury by eliminating intracellular ROS, thus maintaining the normal operation of cells. Additionally, the inhibition of MDA formation demonstrated that RPHs could suppress lipid peroxidation, protect the delicate cell membrane from damage and block reactive oxygen accumulation in the cells, confirming the antioxidant properties. The generally higher efficacy of protection by ERPHs when compared with NRPHs at the same dosage levels may be ascribed to the fact that ERPHs contain more antioxidative peptide fractions^25^. During the same hydrolysis time, the rice protein processed by the electron beam was more likely to be hydrolyzed, producing more polypeptides with antioxidant activity^6^ and thereby effectively protecting the cells against H_2_O_2_-induced oxidative damage.

#### Cell apoptosis

**Figure 5:**
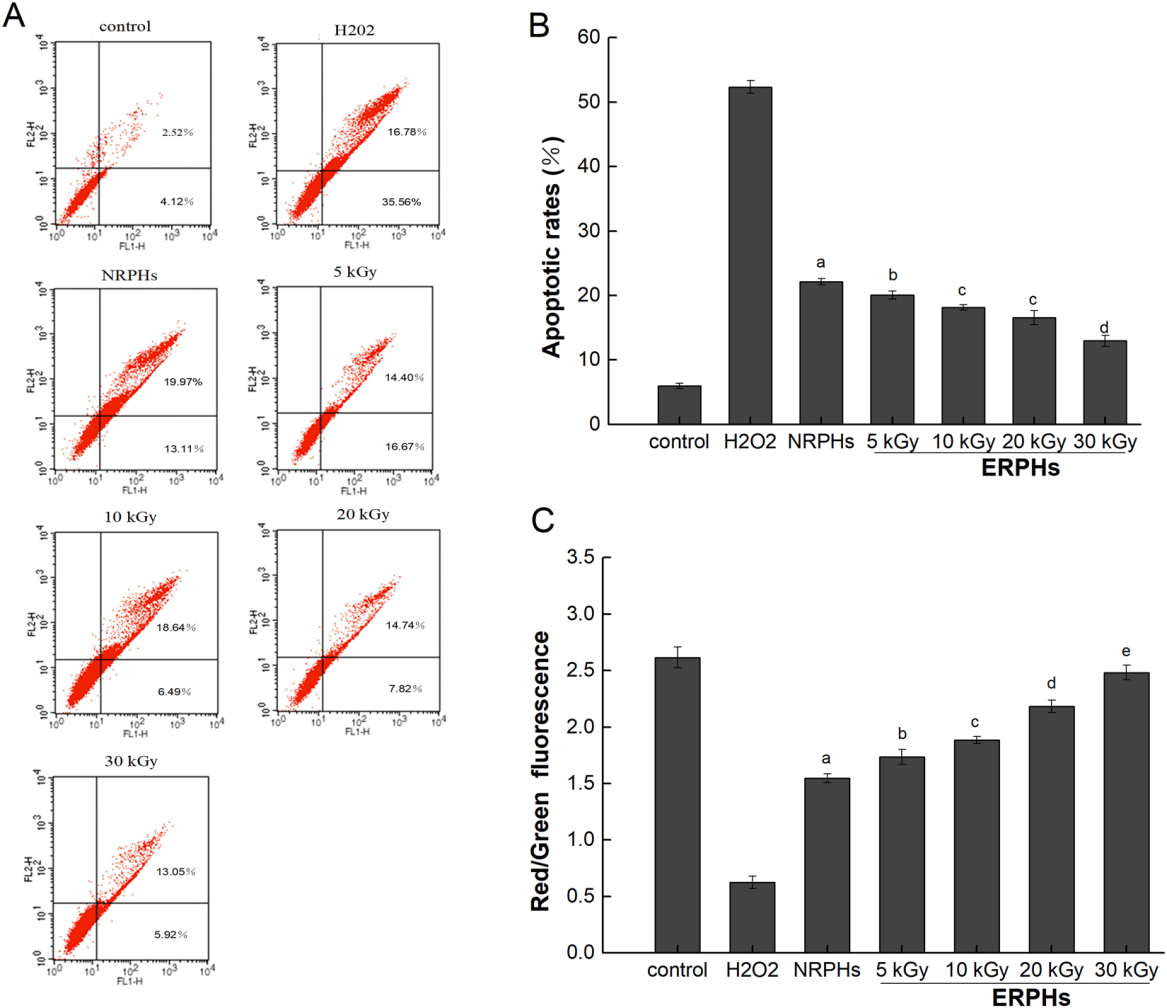
Protective effects of ERPHs against H_2_O_2_-induced apoptosis in HepG-2 cells. The cells were pretreated with NRPHs and ERPHs for 48 h before treated with 0.4 mM H_2_O_2_ for 4 h. Then, cells were me assured by Flow cytometric. (**A**,**B**)Apoptosis detection: Annexin V-FITC assay of HepG-2 cells. (**C**) Alterations of mitochondrial membrane potential (MMP) detect ion: JC-1 assay of HepG-2 cells. Data are shown as means ± S.D.

We examined whether rice protein hydrolysate protected cells through the inhibition of apoptosis. H_2_O_2_-induced apoptosis was determined by an annexin V-FITC/PI assay based on flow cytometry flow. As shown in Fig. 5A, normal cells were double negative and existed in the lower left quadrant. Early apoptotic cells were combined with annexin V-FITC and observed in the lower right quadrant. The cells distributed in the upper right quadrant have been described as advanced apoptotic or necrotic. Figure 5B shows that the percentage of apoptosis increased from 5.98% to 52.34% after exposure to 0.4 mM H_2_O_2_ for 4 h. Preincubation with ERPHs (5, 10, 20 and 30 kGy) for 48 h dose-dependently arrested apoptosis, and the values of apoptosis were decreased to 20.07%, 18.13%, 16.56% and 12.97%, respectively. Moreover, RPHs pretreated with EBI were more efficient than those without modification in protecting HepG-2 cells against H_2_O_2_-induced apoptosis, mainly due to their ability to scavenge more intracellular ROS (Fig. 3A).

Mitochondria are important targets of oxidative damage in cells. The level of mitochondrial membrane potential (MMP) reflects the integrity of the mitochondrial membrane, and the decrease of MMP is therefore considered closely related to mitochondrial dysfunction^26,27^. To estimate the change in the MMP during apoptosis induced by H_2_O_2_ in HepG-2 cells, flow cytometric analysis was carried out using JC-1. The effects of various treatments on the changes in the MMP of HepG-2 cells are shown in Fig. 5C. A marked loss of membrane potential (decrease in the red/green fluorescence intensity ratio) occurred due to oxidative stress induced by exogenous H_2_O_2_. When HepG-2 cells were preincubated with various hydrolysates before apoptotic induction with H_2_O_2_, the loss of membrane potential was significantly restrained. In particular, the MMP of cells pretreated with ERPHs was much higher than that of the NRPH treatment group and exhibited a dose-dependent increase. The 30 kGy preirradiated hydrolysates had the strongest inhibitory effect on the reduction of MMP, with the red/green fluorescence intensity ratio increasing from 0.62 ± 0.05 to 2.48 ± 0.07 compared to that in the H_2_O_2_-induced group. The results indicated that RPHs, especially EBI pretreated rice protein hydrolysates, had the ability to protect the mitochondrial membrane from oxidative damage caused by exogenous hydrogen peroxide.

In summary, the addition of H_2_O_2_ readily damages the microenvironment within the cell, causes oxidative damage to intracellular biomolecules and eventually leads to cell apoptosis^27,28^. On the other hand, excessive ROS accumulated in cells will attack mitochondria and lead to mitochondrial dysfunction, such as the depolarization of the mitochondrial transmembrane potential and release of apoptotic factors, and therefore, the decline in mitochondrial membrane potential is an early event of apoptosis^29^. Our results showed that RPHs can efficiently attenuate H_2_O_2_-induced apoptosis and reduce oxidative damage to the mitochondrial membrane. This positive effect may be because RPHs could effectively remove excess ROS in HepG-2 cells as an antioxidant (Fig. 3A) and decrease oxidative injury, thus inhibiting apoptosis. Furthermore, RPHs can also scavenge ROS by activating antioxidant enzymes in cells, such as SOD, GSH-PX and CAT (Fig. 4A–C), to maintain the balance of the cellular microenvironment and make cells function normally. In addition, the reduction in MDA content within cells indicated that lipid peroxidation was markedly suppressed (Fig. 4D), which guarantees the integrity of the mitochondrial membrane. The exposure of rice protein to an electron beam at 30 kGy prior to alkaline protease treatment may be a good improvement method for the production of peptides with better antioxidative properties. The possible reason is that the electron beam unfolds the structure of the protein and increases the contact area with the enzyme, resulting in more peptides with antioxidant properties.

### Effects of EBI treatment on the structural characteristics of ERPHs

#### Circular dichroism (CD) spectra analysis

To further explore the influence of secondary structure changes on the antioxidant activity of RPHs, CD spectra were used to analyze the content changes of the secondary structure of RPHs before and after EBI treatment, and the results are shown in Table 1. After the enzymatic hydrolysis of rice protein pretreated by EBI, the content of α-helices and β-turns decreased, and the content of random coils increased significantly. Moreover, this change was more obvious with increasing radiation dose. The content of α-helices and β-turns decreased 52.78% and 21.62%, respectively, and the content of random coils increased 14.93% at 30 kGy. This phenomenon suggested that under the action of EBI, the molecular structure of RPHs opened and transformed to an irregular crimp. In addition, a slight increase in the content of β-sheet was observed, indicating that the stretched polypeptide molecules formed β-sheet structures through intermolecular hydrogen bonds. The above results showed that EBI treatment of rice protein transformed the ordered secondary structural units of RPHs into disordered and irregular crimps. This transformation is conducive to exposing more active sites and reducing the steric hindrance effect when interacting with free radicals. Therefore, free radicals can be removed more effective and thus show better antioxidant activity^30,31^.

**Table 1:** Effects of EBI pretreatments on the secondary structure content (%) of ERPHs.

NRPHs		ERPHs			
		5 kGy	10 kGy	20 kGy	30 kGy
α-Helix	10.8 ± 0.37	8.7 ± 0.37	7.2 ± 0.20	6.2 ± 0.34	5.1 ± 0.15
β-Sheet	18.6 ± 0.34	18.9 ± 0.34	19.1 ± 0.19	19.6 ± 0.47	19.9 ± 0.27
β-Turn	14.8 ± 0.17	14.1 ± 0.17	13.8 ± 0.19	12.5 ± 0.40	11.6 ± 0.23
Random roils	55.6 ± 0.51	58.3 ± 0.51	59.2 ± 0.12	61.8 ± 0.36	63.9 ± 0.18

#### Molecular weight (MW) distribution

In this study, RPHs pretreated with EBI were found to exhibit better antioxidant abilities by protecting the cells against H_2_O_2_-induced damage compared to original RPHs. This improvement may be associated with the length of the peptide chain, as it is closely related to the biological activities of hydrolysates^32^. Gel permeation chromatography was used to separate peptide fractions and identify their MW distributions. Table 2 shows the molecular weights of hydrolyzed peptides from rice proteins irradiated at 0–30 kGy. Peptides of <3 kDa accounted for approximately 90% of the hydrolysates, and those with more than 3 kDa made up the remaining hydrolysates. Moreover, a sharp increase was observed in low molecular weight peptides for hydrolysates pretreated with EBI compared with those without modification. The number of small peptides increased as the irradiation dose increased. Hydrolysates preirradiated at 30 kGy contained the highest content of small peptides, with 82.87% of the peptides falling in the range of <1000 Da. For the original hydrolysates, the percentages for 1000 Da fractions were 77.08%, a value much lower than that for ERPHs. It could be proposed that RPs modified by EBI more easily hydrolyzed than un-irradiated sample, generated significantly smaller peptides and thereby greatly enhanced the antioxidant activity of the peptides. This outcome is in agreement with previous report that the size of the peptides is a significant factor in the overall antioxidant activity of hydrolyzed proteins^33^.

**Table 2:** Effects of EBI pretreatments on molecular weight distribution of ERPHs.

Process	NRPHs	ERPHs			
RPHs fractions (%)		5 kGy	10 kGy	20 kGy	30 kGy
>5kD	3.68 ± 0.03	1.88 ± 0.05	1.55 ± 0.12	1.23 ± 0.05	0.46 ± 0.11
3–5 kD	4.06 ± 0.08	2.82 ± 0.11	2.59 ± 0.07	2.18 ± 0.12	0.99 ± 0.15
1–3 kD	15.18 ± 0.13	15.35 ± 0.15	15.47 ± 0.07	15.50 ± 0.11	15.68 ± 0.09
<1 kD	77.08 ± 0.15	79.95 ± 0.09	80.39 ± 0.28	81.09 ± 0.16	82.87 ± 0.08

#### Amino acid composition

The amino acid composition is another crucial factor that can significantly influence the antioxidant properties of protein hydrolysates^17^. Table 3 shows the amino acid composition of rice protein hydrolysates pretreated with or without EBI. The RPHs are rich in Asp, Glu, Arg, Phe, Pro, and Leu, most of which are associated specifically with antioxidant activities either in their free forms or as residues in proteins and peptides^33,34^. Moreover, EBI pretreatment significantly increased the proportion of amino acids related to antioxidation in rice protein hydrolysates, especially Asp, Glu and Leu. However, the increase in the ratio of total antioxidant-related amino acids (TAAA) in hydrolysates had nothing to do with the irradiation dose, resulting in the highest percentage of TAAA (64.61%) at 30 kGy when compared with that in the nonirradiated group (66.39%). The data indicated that the EBI pretreatment of rice protein prior to hydrolysis could facilitate the exposure of antioxidative amino acids and thus improve antioxidant activity. However, the change of TAAA also indicates that the amino acid content is not the decisive factor of the antioxidant activity of the hydrolysates. Many other factors, such as the amino acid sequence of the peptides and molecular weight distribution, together with amino acid content determine the ultimate antioxidant activity of the hydrolysates^35^.

**Table 3:** Effects of EBI pretreatments on amino acid composition (g/100 g of protein) of ERPHs.

Amino acid	NRPHs	ERPHs			
		5 kGy	10 kGy	20 kGy	30 kGy
Asp	8.26 ± 0.17	8.88 ± 0.33	9.4 ± 0.12	9.93 ± 0.19	10.33 ± 0.28
Glu	14.5 ± 0.1	14.87 ± 0.08	15.97 ± 0.04	16.42 ± 0.07	17.44 ± 0.08
Ser	4.03 ± 0.1	3.06 ± 0.04	3.01 ± 0.07	3.33 ± 0.07	3.44 ± 0.1
His	1.95 ± 0.06	2.03 ± 0.19	2.1 ± 0.05	2.3 ± 0.07	2.4 ± 0.14
Gly	4.68 ± 0.05	3.93 ± 0.29	3.78 ± 0.05	3.72 ± 0.06	3.01 ± 0.08
Thr	1.57 ± 0.06	2.65 ± 0.11	3.03 ± 0.1	3.17 ± 0.11	3.26 ± 0.11
Arg	7.12 ± 0.13	7.92 ± 0.75	8.29 ± 0.35	8.46 ± 0.49	8.87 ± 0.33
Ala	4.21 ± 0.13	4.87 ± 0.04	5.09 ± 0.13	5.69 ± 0.04	5.92 ± 0.06
Tyr	2.87 ± 0.17	2.69 ± 0.06	2.99 ± 0.05	3.35 ± 0.09	3.37 ± 0.12
Cys-s	0.49 ± 0.32	0.46 ± 0.16	0.39 ± 0.15	0.24 ± 0.22	0.17 ± 0.32
Val	5.38 ± 0.07	5.61 ± 0.11	5.98 ± 0.14	6.27 ± 0.16	6.39 ± 0.15
met	1.61 ± 0.03	0.91 ± 0.04	0.9 ± 0.08	0.66 ± 0.06	0.66 ± 0.09
Phe	4.28 ± 0.09	4.61 ± 0.06	4.8 ± 0.04	5.16 ± 0.14	5.33 ± 0.07
Ile	3.71 ± 0.1	4.72 ± 0.06	4.92 ± 0.04	5.17 ± 0.04	5.46 ± 0.04
Leu	6.2 ± 0.03	6.82 ± 0.06	7.77 ± 0.05	8.43 ± 0.19	9.4 ± 0.22
Lys	1.88 ± 0.43	3.01 ± 0.19	3.27 ± 0.26	3.58 ± 0.08	3.75 ± 0.27
Pro	3.55 ± 0.07	3.73 ± 0.06	4.16 ± 0.07	4.22 ± 0.13	4.35 ± 0.13
TAAA (%)^a^	64.61	64.93	65.66	65.36	66.39

^a^Total antioxidant related amino acid ratio. Take Asp, Glu, Arg, Phe, Leu and Pro as representatives.

The structural analysis above, combined with the protective effect of the hydrolysates on H_2_O_2-_induced oxidative damage in HepG-2 cells, indicated that the RPHs treated with EBI showed better antioxidant activity, and this improvement was closely related to the change in secondary structure (the conversion of α-helix to random coils). In addition, EBI pretreatment increased the content of antioxidation-related amino acids in the RPHs and produced smaller peptides, thus improving the antioxidant activity of the RPHs. Therefore, EBI can be used as a pretreatment technology to produce peptides with higher antioxidant activity.

## Conclusion

This study demonstrated that the electron beam irradiation process was capable of improving the antioxidant activity of rice protein hydrolysates, which was confirmed by a H_2_O_2_-induced cell damage model. The antioxidative peptides prepared from an electron beam that irradiated rice protein were more efficient for inhibiting H_2_O_2_-induced oxidative damage in human HepG-2 cells, which exhibited stronger antioxidative effects than those without irradiation. The inhibitory mechanism of ERPHs on oxidative damage caused by hydrogen peroxide was the same as that of NRPHs; that is, these molecules can effectively remove the excess ROS in cells and simultaneously activate the antioxidant enzymes in cells and prevent the oxidation of fragile cell membranes, thus ensuring the stability of the cellular microenvironment and inhibiting cell apoptosis. The improvement in antioxidant activity was dose-dependent, and samples preirradiated at 30 kGy showed the strongest protective effect against H_2_O_2_-induced cytotoxicity. On the other hand, CD analysis showed that pretreatment of rice protein with EBIc significantly changed the secondary structure, which is beneficial to the improvement of antioxidative activity. Additionally, the increase in the oxidation resistance of RPHs prepared by proteins irradiated by electron beams indicated that these samples contain more antioxidant-related amino acids and smaller peptides, which, in turn, could effectively eliminate ROS in cells and significantly enhance antioxidant properties. This study provided an efficient way to produce peptides with better antioxidant activity. On the other hand, the EBI irradiation experiment in this paper was conducted in a local factory. The processing capacity of the EBI irradiation equipment depends on the irradiation doses. For instance, the processing capacity is 60 tone per day when the irradiation does is 10 kGy and 30 tone per day when irradiation does is 30 kGy. Thus, the technology in this paper could be applied as industrial scale. However, animal experiment is needed to study the antioxidant activity of irradiated RPHs *in vivo* and whether it has toxic or pathological hazards. And that is what we are focusing on in our next study.
